# Extensive Karyotype Reorganization in the Fish *Gymnotus arapaima* (Gymnotiformes, Gymnotidae) Highlighted by Zoo-FISH Analysis

**DOI:** 10.3389/fgene.2018.00008

**Published:** 2018-01-26

**Authors:** Milla de Andrade Machado, Julio C. Pieczarka, Fernando H. R. Silva, Patricia C. M. O'Brien, Malcolm A. Ferguson-Smith, Cleusa Y. Nagamachi

**Affiliations:** ^1^Laboratório de Citogenética, Centro de Estudos Avançados da Biodiversidade, Instituto de Ciências Biológicas, Universidade Federal do Pará, Belém-Pará, Brazil; ^2^Cambridge Resource Centre for Comparative Genomics, Department of Veterinary Medicine, University of Cambridge, Cambridge, United Kingdom

**Keywords:** chromosome painting, WCP, *Gymnotus*, FISH, cytotaxonomy, karyotype evolution

## Abstract

The genus *Gymnotus* (Gymnotiformes) contains over 40 species of freshwater electric fishes exhibiting a wide distribution throughout Central and South America, and being particularly prevalent in the Amazon basin. Cytogenetics has been an important tool in the cytotaxonomy and elucidation of evolutionary processes in this genus, including the unraveling the variety of diploid chromosome number (2*n* = from 34 to 54), the high karyotype diversity among species with a shared diploid number, different sex chromosome systems, and variation in the distribution of several Repetitive DNAs and colocation and association between those sequences. Recently whole chromosome painting (WCP) has been used for tracking the chromosomal evolution of the genus, showing highly reorganized karyotypes and the conserved synteny of the NOR bearing par within the clade *G. carapo*. In this study, painting probes derived from the chromosomes of *G. carapo* (GCA, 2*n* = 42, 30 m/sm + 12 st/a) were hybridized to the mitotic metaphases of *G. arapaima* (GAR, 2*n* = 44, 24 m/sm + 20 st/a). Our results uncovered chromosomal rearrangements and a high number of repetitive DNA regions. From the 12 chromosome pairs of *G. carapo* that can be individually differentiated (GCA1–3, 6, 7, 9, 14, 16, and 18–21), six pairs (GCA 1, 9, 14, 18, 20, 21) show conserved homology with GAR, five pairs (GCA 1, 9, 14, 20, 21) are also shared with cryptic species *G. carapo* 2*n* = 40 (34 m/sm + 6 st/a) and only the NOR bearing pair (GCA 20) is shared with *G. capanema* (GCP 2*n* = 34, 20 m/sm + 14 st/a). The remaining chromosomes are reorganized in the karyotype of GAR. Despite the close phylogenetic relationships of these species, our chromosome painting studies demonstrate an extensive reorganization of their karyotypes.

## Introduction

*Gymnotus* (Gymnotiformes) is a monophyletic genus of freshwater electric fishes (Albert, 2001; Lovejoy et al., 2010; Tagliacollo et al., 2016) distributed throughout South America (Albert et al., 2005). It represents the most specious genus (40 species; Ferraris et al., 2017) and the widest distribution in the order, with prevalence in the Amazon basin, where several species of *Gymnotus* co-occur in sympatry (Albert and Crampton, 2003; Crampton et al., 2005).

Based on the integrated data from DNA sequencing of six genes, coupled with 223 morphological characters and with Model-Based Total Evidence phylogenetic analyses, Tagliacollo et al. (2016) divided the genus into six clades: *G. pantherinus, G. coatesi, G. anguillaris, G. tigre, G. cylindricus*, and *G. carapo*. The *Gymnotus carapo* group is regarded as monophyletic and is located in a derived position within the genus (Albert, 2001; Lovejoy et al., 2010; Tagliacollo et al., 2016). Craig et al. (2017) described seven subspecies for *G. carapo*.

**Figure 1:**
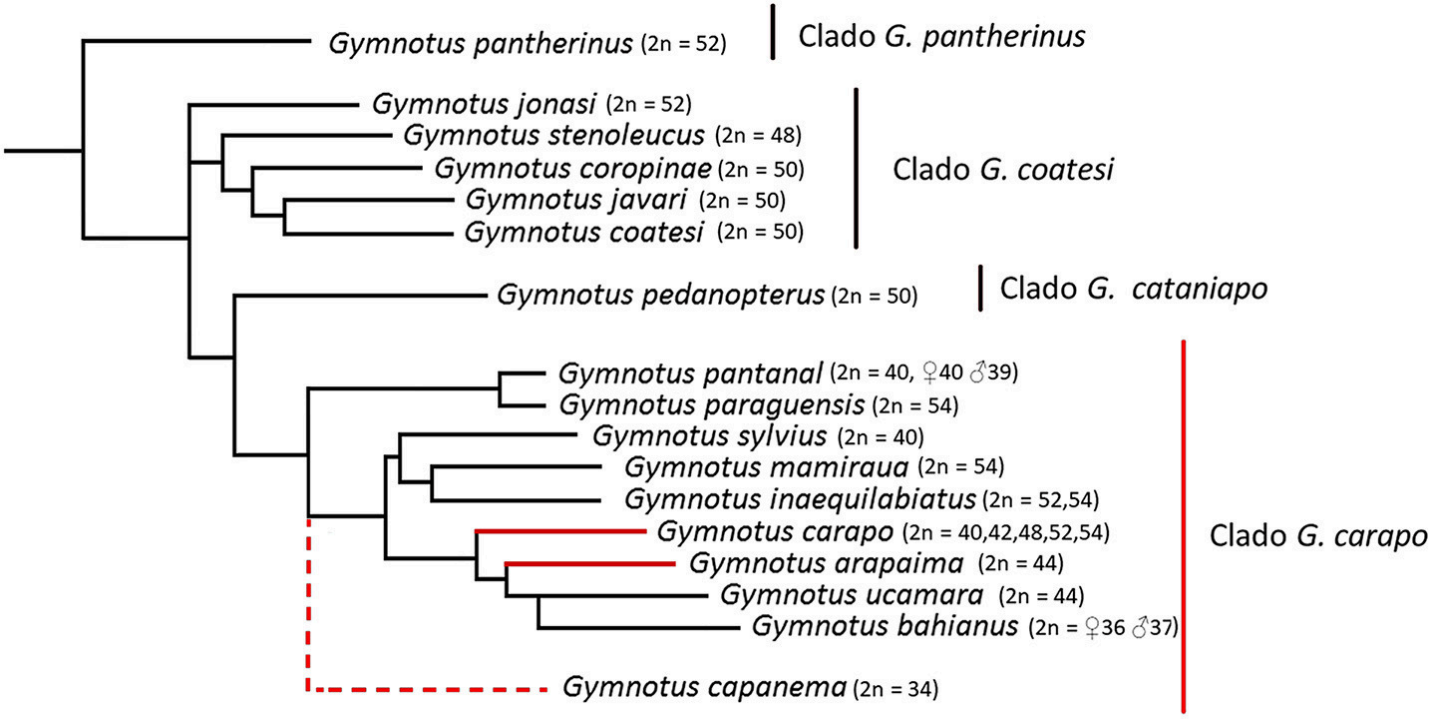
Representative tree of species of *Gymnotus* with diploid number known (Data present in Table 1). It was included only species with know phylogenetic relationships, based on data from Albert et al. (2005) and Tagliacollo et al. (2016). *G. capanema* was included in the *G. carapo* clade based on Milhomem et al. (2012a), but has unclear place within the clade.

Cytogenetics has been an important tool in cytotaxonomy and has proved to be very useful in understanding the evolutionary processes behind the diversification of *Gymnotus*. The Gymnotiformes order has considerable variation, not only in diploid number (from 2*n* = 24 in *Apteronotus albifrons*, Howell, 1972; Almeida-Toledo et al., 1981; Mendes et al., 2012; to 2*n* = 74 in *Rhabdolichops* cf *eastward*, Suárez et al., 2017) but also in the karyotype formula and location of repetitive sequences (Fernandes et al., 2005; Almeida-Toledo et al., 2007; Silva et al., 2009; da Silva et al., 2013; Jesus et al., 2016; Araya-Jaime et al., 2017; Batista et al., 2017; Sousa et al., 2017; Takagui et al., 2017). Recently, fluorescence *in situ* hybridization (FISH), has played an important role in understanding the genome structure of fish species (Yi et al., 2003; Cabral-de-Mello and Martins, 2010; Martins et al., 2011; Vicari et al., 2011; Gornung, 2013; Knytl et al., 2013; Yano et al., 2017) and molecular cytogenetic studies in Gymnotiformes have shown dynamic reorganization, including pericentric inversions observed through repetitive DNA position (Fernandes et al., 2017), sequence dispersion via transposable elements and the association between different repetitive sequences (Utsunomia et al., 2014; da Silva et al., 2016; Machado et al., 2017) and the presence of different sex chromosome systems (Margarido et al., 2007; Henning et al., 2008, 2011; da Silva et al., 2011, 2014; Almeida et al., 2015). This evolutionary plasticity of the karyotype is seen in *Gymnotus* (Table 1), a genus that has high interspecific variability in chromosome numbers (Figure 1, Table 1), ranging from 2*n* = 34 in *Gymnotus capanema* (Milhomem et al., 2012a) to 2*n* = 54 in *G. carapo* (Foresti et al., 1984), *G. mamiraua* (Milhomem et al., 2007), *G. paraguensis* (Margarido et al., 2007) and *G. inaequilabiatus* (Scacchetti et al., 2011). *Gymnotus arapaima* is located within the *G. carapo* clade, with 2*n* = 44 (24 m/sm + 20 st/a; Milhomem et al., 2012b).

**Table 1:** Cytogenetic data from the genus *Gymnotus*, including 2*n*, karyotype formula (KF), NOR and ribosomal DNA sequences 18 and 5S.

**Species**	**2*n* (KF)**	NOR^*^	18S^*^	5S^*^	**Authors**
*Gymnotus arapaima*	44 (24 m/sm + 20 st/a)	2	2	–	Milhomem et al., 2012b
*Gymnotus bahianus*	♀36 (30 m/sm + 6 st) ♂37 (32 m/sm + 5 st)	2	2	2	Almeida et al., 2015
*Gymnotus carapo*	54 (54 m/sm)	2	–	–	Foresti et al., 1984
	52 (50 m/sm + 2 st/a)	2	–	–	
	48 (34 m/sm + 14 st/a)	–	–	–	
	42 (32 m/sm + 10 st/a)	2	–	–	Fernandes-Matioli et al., 1998
	54 (52 m/sm + 2 st/a)	2	–	–	Claro, 2008
	54 (52 m/sm + 2 st/a)	2	14	2	Milhomem et al., 2007
	42 (30 m/sm + 12 st/a)	2	–	–	Milhomem et al., 2008
	40 (28 m/sm + 12 st/a)	2	–	–	
*Gymnotus* cf. *carapo*	54 (50 m/sm + 4 st/a)	2	2	≤ 30	Scacchetti et al., 2011
*Gymnotus carapo'Catalão'*	40 (30 m/sm + 10 st)	–	2	4	da Silva et al., 2014
*Gymnotus carapo'Maranhão'*	42 (30 m/sm + 12 st/4a)	–	2	14	da Silva, 2015
*Gymnotus capanema*	34 (20 m/sm + 14 st/a)	2	2	–	Milhomem et al., 2012a
*Gymnotus coatesi*	50 (24 m/sm + 26 st/a)	8	19	2	Machado et al., 2017
*Gymnotus coropinae*	♀50 (28 m/sm + 22 st/a) ♂49 (26 m/sm + 23 st/a)	–	2	2	da Silva et al., 2014
*Gymnotus inaequilabiatus*	52 (50 m/sm + 2 st/a)	2	–	–	Fernandes-Matioli et al., 1998
	54 (52 m/sm + 2 st/a)	–	2	≤34	Scacchetti et al., 2011
*Gymnotus javari*	50 (20 m/sm + 30 st/a)	–	–	2	Utsunomia et al., 2014
*Gymnotus jonasi*	52 (12 m/sm + 40 st/a)	6	6	–	Milhomem et al., 2012b
*Gymnotus mamiraua*	54 (50 m/sm + 4 st/a)	–	–	–	
	54 (38 m/sm + 16 st/a)	2	2	26	Milhomem et al., 2012b da Silva et al., 2016
*Gymnotus pantanal*	40 (14 m/sm + 26 st/a) ♀40 (14 m/sm + 26 st/a) ♂39 (15 m/sm + 24 st/a)	4 2	– –	– 4	Fernandes et al., 2005. Margarido et al., 2007; da Silva et al., 2011
*Gymnotus pantherinus*	52 (46 m/sm + 6 st/a)	2	–	–	Fernandes-Matioli et al., 1998
	52 (50 m/sm + 2 st/a)	2	2	4	Scacchetti et al., 2011
*Gymnotus paraguensis*	54 (52 m/sm + 2 st)	2	–	38	Margarido et al., 2007; da Silva et al., 2011
	54 (50 m/sm + 4 st)	2	–	–	Lacerda and Maistro, 2007
*Gymnotus* cf. *pedanopterus*	50 (42 m/sm + 8 st/a)	–	2	2	da Silva, 2015
*Gymnotus* cf. *stenoleucus*	48 (20 m/sm + 28 st/a)	–	2	2	da Silva, 2015
*Gymnotus sylvius*	40 (38 m/sm + 2 st/a)	2	–	–	Fernandes-Matioli et al., 1998
	40 (30 m/sm + 10 st/a)	2	–	–	Albert et al., 1999
	40 (38 m/sm + 2 st/a)	2	–	–	Claro, 2008
	40 (36 m/sm + 4 st/a)	2	–	–	Lacerda and Maistro, 2007
	40 (36 m/sm + 4 st/a)	2	–	–	Margarido et al., 2007
	40 (34 m/sm + 6 st)	2	2	2	Scacchetti et al., 2011
*Gymnotus ucamara*	44 (28 m/sm + 16 st/a)	–	2	4	da Silva, 2015
*Gymnotus* sp.	50 (26 m/sm + 24 st/a)	2	–	–	Lacerda and Maistro, 2007
*Gymnotus* sp. ‘Negro’	♀50 (22 m/sm + 28 st) ♂50 (21 m/sm + 29 st)	–	2	4	da Silva et al., 2014

* *Number of chromosome with signals; m, metacentric; sm, submetacentric; st, subtelocentric; a, acrocentric*.

Whole chromosome painting (WCP) techniques use specific painting probes of whole chromosomes, chromosomes arms or chromosome regions to find homologous segments in other species (Yang and Graphodatsky, 2017) and Nagamachi et al. (2010) produced whole chromosome probes from *G. carapo* (GCA, 2*n* = 42) by chromosome sorting using flow cytometry and made a comparative genomic map against the chromosomal background of the cytotype with 2*n* = 40 chromosomes. The results uncovered a high degree of chromosomal repatterning between these cytotypes, with only eight pairs showing conserved synteny (GCA 1, 2, 6, 9, 14, 19, 20, 21). Nagamachi et al. (2013) used the same set of probes for *G. capanema* (GCP, 2*n* = 34) and the results showed that the degree of genomic reorganization was much higher, with only four pairs (GCA 6, 7, 19, 20) showing conserved synteny with GCA 2*n* = 42 and three pairs (GCA 6, 19, 20) with GCA 2*n* = 40. Of these, GCA 7 and 19 are associated with other chromosomes in the karyotype of GCP. The study of Milhomem et al. (2013), with the probe derived from the NOR bearing par of GCA, 2*n* = 42, shows that there is a possible synapomorphy of the NOR bearing par within the *G. carapo* clade.

We use the same set of probes produced by Nagamachi et al. (2010) to analyze the karyotype of *G. arapaima* and to compare the results with our previous studies of species in the genus *Gymnotus*. Our findings confirm and extend our understanding of the extensive karyotype reorganization within this genus.

## Materials and methods

### Sampling

Samples of *G. arapaima* (GAR, 2*n* = 44, 24 m/sm + 20 st/a) were collected in the Mamiraua Reserve (Reserva de Desenvolvimento Sustentável Mamiraua) in the Amazon basin, Brazil (03°02′11.8″S 064°51′16.6″W). These samples were previously analyzed by conventional cytogenetic methods (Milhomem et al., 2012b). The animals collected were handled following procedures recommended by the American Fisheries Society. JCP has a permanent field permit, number 13248 from “Instituto Chico Mendes de Conservação da Biodiversidade.” The Cytogenetics Laboratory of UFPa has permit number 19/2003 from the Ministry of Environment for sample transport and permit 52/2003 for using the samples for research. The Ethics Committee of the Federal University of Para (Comitê de Ética Animal da Universidade Federal do Pará) approved this research (Permit 68/2015).

### WCP

**Figure 2:**
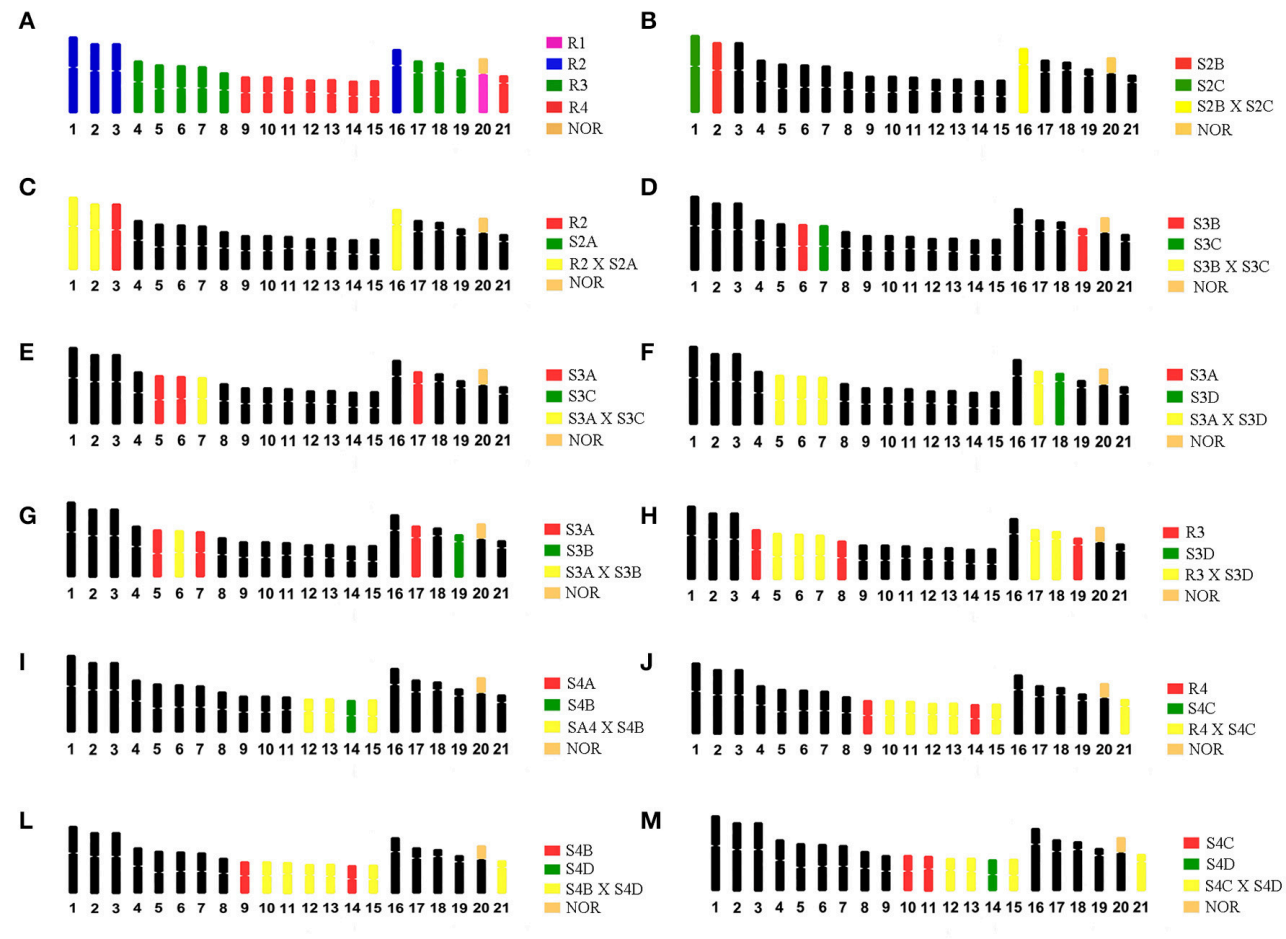
Ideograms of the karyotype of *G. carapo* (2*n* = 42) representing: **(A)** The four chromosome regions (R1, R2, R3, R4) obtained by Nagamachi et al. (2010). **(B–M)** Dual – color FISH experiments based on Nagamachi et al. (2010, 2013), to identify chromosomal homology with GCA (2*n* = 42). S2A, S2B, and S2C represents sub-regions **(A–C)** within region 2; S3A, S3B, S3C, and S3D represents sub-regions **(A–D)** within region 3; S4A, S4B, S4C, and S4D represents sub-regions **(A–D)** within region 4.

WCP probes from *G. carapo* (2*n* = 42; 30 m/sm + 12 st/a) described in Nagamachi et al. (2010) were hybridized onto metaphases of *G. arapaima* (GAR, 2*n* = 44, 24 m/sm + 20 st/a). The chromosomes of GCA, 2*n* = 42 were flow-sorted into four regions (R1–R4), from which probes were produced. R1 represented the NOR-bearing chromosome (GCA20), R2 contains the four largest pairs (1–3 and 16); R3 contains the eight medium-sized pairs (4–8 and 17–19) and R4 the eight smallest pairs (9–15 and 21). Additional sorting produced subregion probes (S) from each of the three regions with multiple chromosome pairs included (R2, R3 and R4). R2: S2A (GCA 1, 2 and 16); S2B (GCA 2 and 16) and S2C (GCA 1 and 16). R3: S3A GCA (5–7 and 17); S3B (GCA6 not 7; re-analyzed in Nagamachi et al., 2013, GCA 19); S3C (GCA 7); and S3D (GCA5–7, 17 and 18). R4: S4A (GCA 12, 13 and 15); S4B (GCA 12–15); S4C (GCA 10–13, 15 and 21); and S4D (GCA 12–15 and 21). For details, see Figure 2 and Table 1 in Nagamachi et al. (2010).

To find out the corresponding segments between GAR and GCA (2*n* = 42), we used dual-color FISH with probes from R3 and R4. The other non-hybridized chromosomes or segments correspond to R1 (GAR 19, Milhomem et al., 2013) or R2. For a more refined identification of the chromosomes from R2, R3 and R4, we employed dual-color FISH using probes from the subregions as specified in Table 2 of Nagamachi et al. (2010), with some modifications related to the identification of the chromosomes of S3B made in Nagamachi et al. (2013). With those experiments (as illustrated in Figure 2) it was possible to identify individually GCA pairs 1–3, 6, 7, 9, 14, 16, and 18–21, while it was not possible to distinguish the pairs [4, 8], [10, 11], [5, 17], and [12, 13, 15].

**Table 2:** Chromosome homologies between *G. carapo* (2*n* = 42), *G. carapo* (2*n* = 40), *G. capanema* (2*n* = 34), and *G. arapaima* (2*n* = 44).

**Region**	***G. carapo* chromosome**		***G. capanema* chromosome**	***G. arapaima* chromosome**
	**GCA, 2*n* = 42^a^**	**GCA, 2*n* = 40^a^**	**GCP, 2*n* = 34^b^**	**GAR, 2*n* = 44**
R 1	20	20	15	19
R 2	1	1	5q + 9q	1
	2	2	3q_dist_ + 16	14q_dist_ + 21
	3	5q_dist_ + 6 (p + q_prox_)	2p_dist_ + 12q_dist_ + 13q_prox_	13q_dist_ + 18
	16	7q + 18 (p + q_prox_)	7q + 14	2 + 14q_prox_
R 3	[4, 8]	6q_dist_ + 9q + 10	4q_dist_ + 6p	5+ 20
	6	11	8	4q + 16q_int_
	7	8p + 9p	1q_dist_	3 + 16p
	[5, 17]	4, 8q, 18q_dist_	9p + 11 + 12 (except q_dist_)	6 + 16q (except q_int_)
	18	3p + 7p	1p_prox_ + 2p_prox_ + 4q_prox_	15
	19	19	2q_dist_	7p + 22
R 4	9	14	7p + 3q_prox_	8
	[10, 11]	5p + q_prox_, 12q	10 + 17	4p + 7q + 12
	[12, 13, 15]	3q, 12p, 13, 16	1p_dist_ + 2q_prox_ + 6q_dist_	10 + 11 + 13 (p + q_prox_)
	14	17	13p + 13q_dist_	9
	21	15	6q_prox_ + 1q_prox_	17

_dist_, distal; _prox_, proximal; _int_, interstitial.

a According to Nagamachi et al. (2010).

b *According to Nagamachi et al. (2013)*.

### FISH

Chromosome painting techniques followed Yang et al. (1995) with adaptations. Slides were digested with 1% pepsin to remove the excess of cytoplasm, treated with formaldehyde 1%, and dehydrated in ethanol series (2x 2 min 70%, 2x 2 min 90%, and 1x 4 min 100%). Subsequently the slides were aged overnight at 37°C. The probes were prepared following Nagamachi et al. (2010), denatured for 15 min at 70°C and applied onto a slide with chromosomes that were previously denatured at 70°C for 4 min in 70% formamide/2× SSC [pH 7.0]. The hybridization lasted 72 h at 37°C. The slides were washed once in a solution of 50% formamide/2× SSC, once in 2× SSC and once in 4× Tween, 5 min each.

The dual-color FISH experiments were made with probes that were either directly labeled or biotinylated detected with avidin, (Vector Laboratories, Burlingame, CA, USA) linked to Cy3 or FITC (Amersham, Piscataway, NJ, United States). DAPI (4′,6-diamidino-2-phenylindole) was used as a counterstain.

### Microscopy and image processing

Image acquisition was made using the software Nis-elements in the microscope Nikon H550S. Chromosomes were morphologically classified according to Levan et al. (1964). The karyotype was organized according to Milhomem et al. (2012b).

## Results

**Figure 3:**
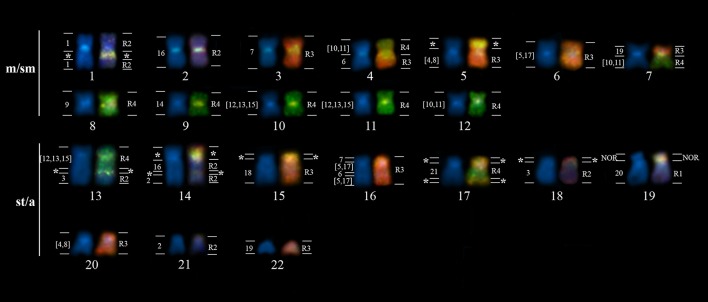
Haploid karyotype of *G. arapaima* (GAR) arranged from mitotic chromosomes after dual-color hybridization with probes derived from Region 3 (R3, red) and Region 4 (R4, green) from the *Gymnotus carapo* (GCA) chromosome complement. Regions R1 and R2 were not subjected to FISH analysis and, therefore, the equivalent homeologous parts on GAR chromosomes are DAPI-stained (blue) only. For each of the 22 GAR chromosome pairs, the DAPI-only stained homolog is depicted on the left, while the dual-color FISH hybridization pattern is present on the right. The correspondence to *G. carapo* (GCA) homeologous chromosomes is indicated by chromosome pair numbers on the left side of the DAPI-stained GAR chromosomes, while the correspondence to the particular GCA regions (R1–4) is indicated on the right side of FISH-painted chromosomes. ^*^Repetitive sequences.

The whole chromosome probes from *G. carapo* were hybridized to chromosomes of *G. arapaima*. The regions of homology (hereafter designated as R1-4) obtained with GCA (2*n* = 42) probes against the chromosomes of GAR are indicated on the karyotype of GAR arranged from DAPI-stained chromosomes (Figure 3). Dual color FISH with the probes of R3 (red) and R4 (green) defined the chromosome groups in GAR that corresponded to the four groups of regions in GCA (Figure 3), as R3 and R4 do not share chromosome pairs. Any chromosome segments hybridizing simultaneously with two colors indicate repetitive DNA sequences that are common to both regions. The chromosomes or segments in blue (DAPI) represent the NOR-bearing chromosomes (R1, GCA20) and the chromosomes corresponding to R2 (pairs 1–3 and 16). Table 2 shows the correspondence of the GCA (2*n* = 42) chromosomes with the previously published karyotypes of GCA (2*n* = 40) and GCP (2*n* = 34), and GAR (2*n* = 44, present study).

From the 12 chromosome pairs of *G. carapo* that can be individually differentiated (GCA 1–3, 6, 7, 9, 14, 16, and 18–21), six pairs (GCA 1, 9, 14, 18, 20, 21) have conserved homology within GAR. GCA 20 hybridizes to one whole chromosome, pair 19, as described by Milhomem et al. (2013). Six chromosome pairs (GCA 2, 3, 6, 7, 16, and 19) show two signals on GAR chromosomes.

The GCA probes that represent two chromosome pairs [4, 8] revealed two signals, and pairs [10, 11] and [5, 17] revealed three signals and the probe representing three pairs [12, 13, 15] also revealed three signals on GAR chromosomes.

The following associations were found: GAR 4: [10, 11]/C/6, GAR 7: 19/C/[10, 11], GAR 13: [12, 13, 15]/C/ [12, 13, 15]/^*^/3, GAR 14: ^*^/C/16/^*^/2, GAR 16: 7/C/ [5, 17]/6/ [5, 17] (where C = centromere and ^*^ = repetitive sequences).

## Discussion

Our results demonstrate that the genomic reorganization in the analyzed species of *Gymnotus* is greater than that assumed by classical cytogenetics (Milhomem et al., 2008, 2012a,b).

**Figure 4:**
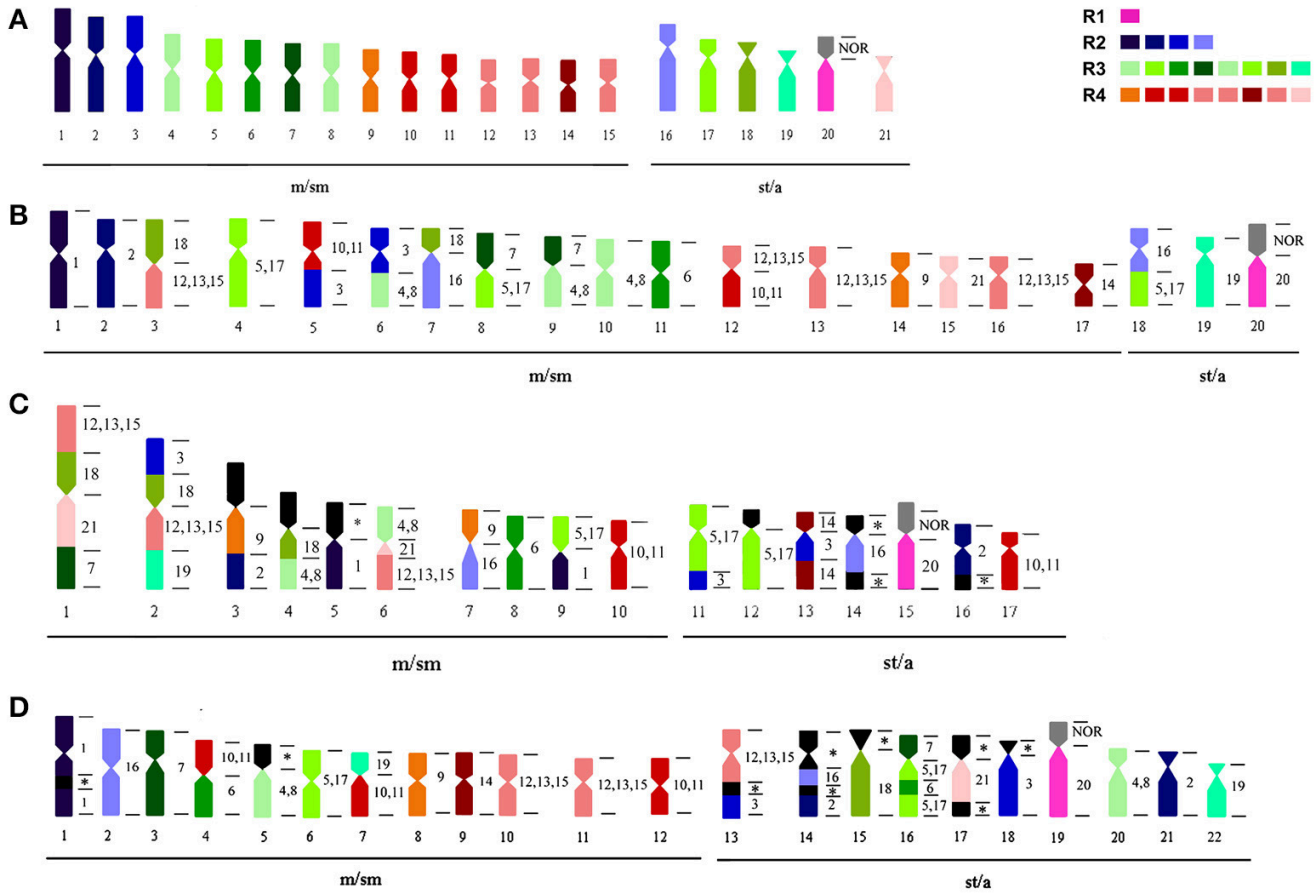
Ideogram with the karyotypes of **(A)** *G. carapo* (2*n* = 42); **(B)** *G. carapo* (2*n* = 40); **(C)** *G. capanema*, and **(D)** *G. arapaima*. The numbers at the right side of chromosomes in **(B–D)** show the homology with the karyotype **(A)** of *G. carapo*. Each color in the karyotypes **(B–D)** represents the correspondent chromosome colored in **(A)**. Chromosomes groups [4, 8]; [5, 17]; [10, 11], and [12, 13, 15] share the same color within each group.

Whole chromosome probes from GCA 2*n* = 42 have been used for comparative genomic mapping (CGM) of the karyotype of (i) cryptic species GCA 2*n* = 40 (Nagamachi et al., 2010), (ii) GCP 2*n* = 34 (Nagamachi et al., 2013) and, in the present work, iii) onto the karyotype of GAR 2*n* = 44 (Figure 4). Similar to the observations in the two previously mapped species (Nagamachi et al., 2010, 2013), GAR also presents a highly reorganized karyotype (Figures 3, 4, Table 2) in relation to GCA 2*n* = 42 and also in relation to GCA 2*n* = 40 and GCP 2*n* = 34. From the 12 chromosome pairs of GCA 2*n* = 42 that can be individually differentiated (GCA 1–3, 6, 7, 9, 14, 16, 18–21), GAR shows conserved synteny of six pairs (GCA 1, 9, 14, 18, 20, 21); five pairs (GCA 1, 9, 14, 20, 21) with the cryptic species GCA 2*n* = 40 and only one pair with GCP (GCA 20). On the other hand, GCA 2*n* = 40 shares with GCA 2*n* = 42, eight pairs (GCA 1, 2, 6, 9, 14, 19, 20, 21) and with GCP, three pairs (GCA 6, 19, 20) (Figure 4). It is also worth noting that the probes representing GCA [4, 8] and [12, 13, 15] show two and three signals, respectively, in three species (GCA 42, GCP and GAR) indicating that these chromosomes may have retained their homology.

A comparative analysis of the WCP data described above shows that the karyotypes of both GCP and GAR are related to the karyotypes of GCA. GCP, although part of the *carapo* group (Milhomem et al., 2012a), has an uncertain position inside the phylogeny of the clade, while GCA and GAR are closely related. GCP and GAR do not share the same chromosome rearrangements (Table 2), meaning that these rearrangements must have occurred after their speciation. The results of the CGM suggest either a divergence prior to that of GAR or a recent divergence characterized by fast karyotype evolution and fixation of a high number of chromosomal rearrangements.

**Figure 5:**
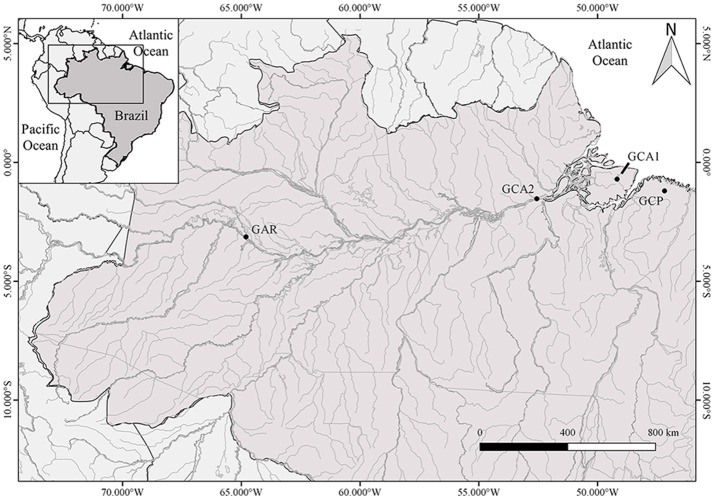
A map of Northern Brazil showing the geographical distribution of the samples from the four species of *Gymnotus* analyzed by whole chromosome painting. *G. carapo* 2*n* = 42 (GCA1, Nagamachi et al., 2010, Santa Cruz do Arari, Marajo Island), *G. carapo* 2*n* = 40 (GCA2, Nagamachi et al., 2010, Almerim, Amazon river drainage), *G. capanema* (GCP, Nagamachi et al., 2013, Capanema, Rio Açaiteuazinho drainage) and *G. arapaima* (GAR, present study, Mamirauá Sustainable Development Reserve, Amazonas, Brazil).

It is also clear that the karyotype of GAR is evolutionary closer to the GCA karyotype than to the GCP karyotype. However, GAR is located 2000 km away from the other species, while GCP and GCA (2*n* = 42) are 200 km apart (Figure 5). This might suggest that the karyotypes of GCA and GAR are more conserved while GCP changed over a shorter period of time. Another explanation for this huge differentiation of the GCP karyotype might lie in the fact that this species inhabits Rio Açaiteuazinho drainage from Northeast Para, which is not connected with the Amazon basin, while GCA and GAR are part of the same hydrographic basin, despite the long distance between them (Figure 5).

Freshwater fishes in general have a higher rate of chromosomal rearrangements than marine fishes due to the reduced flow with the natural barriers present in the freshwater environment compared to the open marine biome, with bigger populations and high potential for dispersion and higher gene flow, reducing the chance for karyotype changes to fixate in the population (Molina, 2007; Nirchio et al., 2014; Artoni et al., 2015). Lande (1977) theorizes that the rates of chromosomal rearrangement are proportional to selection and inversely proportional to the effective size of the population and Araya-Jaime et al. (2017) suggests that this could be considered a general model of chromosomal evolution within Gymnotiformes, since populations with little or no geneflow may facilitate the fixation of chromosomal rearrangements within a particular species in a shorter evolutionary time. This may be a contributory factor to speciation within the group and may also contribute to the higher number of rearrangements found. It is a valid reminder that the high number of rearrangements observed in the present study was possible through WCP, and groups with a more stable diploid number and karyotypic formula potentially could have fixed a higher number of rearrangements that did not cause major structural changes.

As Region 3 was labeled with a red fluorochrome and Region 4 with a green one, all yellow regions in Figure 3 are the result of hybridization of both probes to the same region. Although R3 and R4 do not share the same chromosome pair, they share the same or highly similar repetitive DNA. The hybridization of both probes to the same regions of GAR chromosomes confirms that this sequence is also present in this species. Since repetitive sequences evolve quickly by concerted evolution with significant differences between species (Pons and Gillespie, 2004), the presence of the highly similar repetitive DNA sequence in different species clearly shows that these species diverged recently, without sufficient time to accumulate sequence differences. Despite the huge amount of rearrangement, the repetitive DNA sequence strongly suggests that these species diverged recently and also that the rearrangements responsible for the karyotypic differences are also recent.

Taken together, the sum of the results might explain the difficulty in finding synapomorphies among the species compared so far, since most of the rearrangements might have become fixed after the species became isolated. On the other hand, because the *G. carapo* clade is a derived one (Tagliacollo et al., 2016, Figure 1) and because up until today there are few species of *Gymnotus* studied by chromosome painting, we currently cannot conclusively resolve whether the homologous chromosomes present a symplesiomorphic or synapomorphic character. An example is the NOR bearing pair that maps to GCA 20 using rDNA probes in species of the *carapo* group, but this location is different in species outside this group (Milhomem et al., 2013), which suggests that it is a synapomorphy. This matter will be better understood once species outside the *carapo* group are mapped with all the GCA whole chromosome probes.

## Author contributions

MM, JP, FS, PO, MF-S, and CN: gave substantial contributions to the conception of the work; the acquisition, analysis, and interpretation of data for the work; participated in the draft of the work or revised it critically for important intellectual content; gave final approval of the version to be published; and agreed to be accountable for all aspects of the work in ensuring that questions related to the accuracy or integrity of any part of the work are appropriately investigated and resolved.

### Conflict of interest statement

The authors declare that the research was conducted in the absence of any commercial or financial relationships that could be construed as a potential conflict of interest.
